# Building local capacity for cervical cancer prevention in low resource settings: Changing strategy during the COVID-19 pandemic

**DOI:** 10.7189/jogh.11.03044

**Published:** 2021-03-01

**Authors:** Mila P Salcedo, Melissa L Varon, Natacha Phoolcharoen, Nafissa Osman, Ernestina David, Ricardina Rangeiro, Dercia Changule, Viviane Andrade, Andrea Neves, Kathleen M Doughtie, Jennifer Carns, Cesaltina Lorenzoni, Ellen Baker, Kathleen M Schmeler

**Affiliations:** 1 The Obstetrics and Gynecology Department, Federal University of Health Sciences of Porto Alegre (UFCSPA)/ Santa Casa de Misericordia Hospital of Porto Alegre, Porto Alegre, Brazil; 2 The Department of Gynecologic Oncology & Reproductive Medicine, The University of Texas MD Anderson Cancer Center, Houston, Texas, USA; 3 Department of Obstetrics and Gynecology, King Chulalongkorn Memorial Hospital, Bangkok, Thailand; 4 Hospital Central de Maputo, Maputo, Mozambique; 5 Universidade Eduardo Mondlane (UEM), Maputo, Mozambique; 6 Ministerio da Saude de Moçambique (MISAU), Maputo, Mozambique; 7 Hospital de Câncer de Barretos, Barretos, Brazil; 8 Hospital Geral José Macamo, Maputo, Mozambique; 9 The Department of Breast Radiation Oncology, The University of Texas MD Anderson Cancer Center, Houston, Texas, USA; 10 Department of Bioengineering, Rice University, Houston, Texas, USA

In low- and middle-income countries (LMIC), where the great majority of cervical cancer cases occur, there is a shortage of health care providers trained to diagnose and treat pre-invasive cervical disease. The cervical cancer regional incidence and mortality rates are highest in sub-Saharan Africa and South-Eastern Asia [1]. In many resource-constrained regions, the shortage of trained providers limits the scale-up of quality cervical cancer screening, diagnosis and treatment services. In Mozambique, cervical cancer is the primary cause of cancer and cancer-related deaths among women [2,3]. Since 2016 we have provided in-person support and training to gynecologists and nurses in Mozambique. Cervical cancer prevention training, included teaching skills of colposcopy, cervical biopsy and loop electrosurgical excision procedure (LEEP) [4] Completion of hands-on training was followed by patient care with the trainers in local clinics. Participation in monthly Project ECHO (Extension of Community Healthcare Outcomes) telementoring sessions was encouraged to reinforce and amplify knowledge and skills. Since March 2020 travel has been restricted due to coronavirus disease (COVID-19). We have therefore adapted the way we deliver this training and provide support to colleagues in Mozambique so that capacity building efforts continue.

## PRE-INVASIVE CERVICAL DISEASE TRAINING

**Figure 1:**
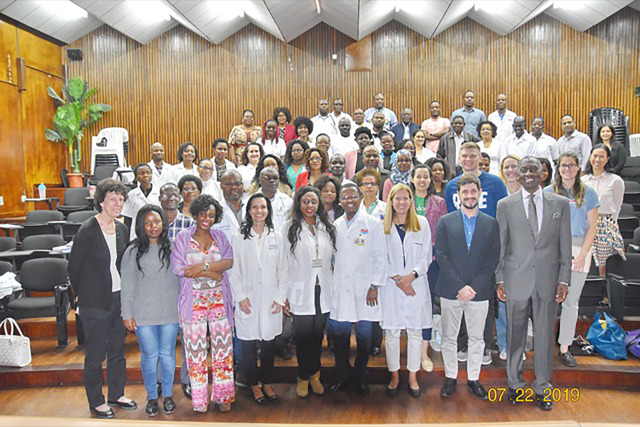
Pre-invasive cervical disease training course participants.

**Figure 2:**
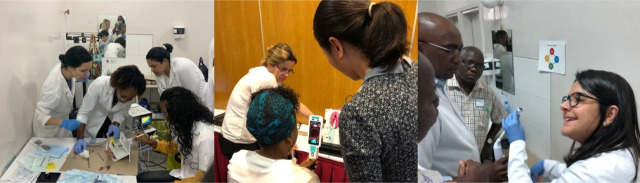
Pre-invasive cervical disease training course stations.

Since 2016 our team of physicians, nurses and public health professionals has delivered cervical cancer prevention training to health care providers in Texas, Latin America, Africa and Nepal. These 1- to 3-day training courses include lectures and skills based training in colposcopy, cervical biopsy and LEEP (**Figure 1**). We partner regionally with local clinics, hospitals, ministries of health and nonprofit organizations. Training offered is part of a comprehensive strategy to increase local capacity to provide and access these services. Lectures focus on the natural history of cervical cancer and its link to the human papillomavirus (HPV), HPV vaccination, cervical cancer screening options and strategies, and diagnosis and treatment of pre-invasive cervical disease. Courses are developed in collaboration with local partners and consider local resources to meet the needs of the course attendees. Hands-on training, divided into four to six stations, is customized to accommodate local needs and may include: visual inspection with acetic acid (VIA), thermal ablation, cryotherapy, colposcopy, cervical biopsy, endocervical curettage and LEEP (**Figure 2**). Following the course, we accompany a smaller group of attendees for 1-2 days to their clinics to provide guided supervision in the delivery of patient care. The training uses high fidelity, innovative models developed by bioengineers from Rice University. These models, Low-cost Universal Cervical Cancer Instructional Apparatus (LUCIA), provide a good platform for teaching VIA, colposcopy, cervical biopsy, ablation and LEEP [5].

Using the Project ECHO model, telementoring extends training and keeps providers engaged in learning. During monthly ECHO videoconferences, local clinicians present patient cases for discussion with specialists and their peers.

Since 2015, we have delivered 19 courses in seven countries (El Salvador, Mozambique, Trinidad and Tobago, Lesotho, Malawi, Nepal and United States of America). In Mozambique we have held twelve courses.

## PROGRAM ADAPTATIONS DURING THE COVID-19 PANDEMIC

**Figure 3:**
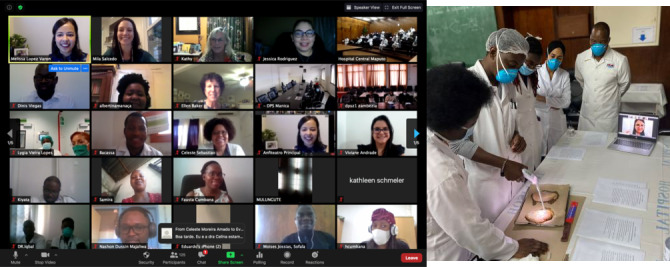
Virtual course (from the author’s own collection (used with permission).

Due to travel restrictions during the pandemic training courses have been transitioned to a blended virtual platform. In July and August 2020 two courses were held. More than 150 health care providers from five countries (Mozambique, Angola, Zambia, Brazil and Portugal) attended online lectures and 18 clinicians also received skills training in-person. International faculty attended virtually, providing some lectures, and virtual guidance and support during skills training as needed (**Figure 3**). The majority of attendees connected remotely for the lectures. Eighteen attendees were trained on-site by local gynecologists at the skills stations, with US and Brazilian faculty observing virtually. Skills-based training included colposcopy, cervical biopsy, endocervical curettage, thermal ablation and LEEP. The planning for this format included a revision of the lecture components encouraging participant involvement by using polling questions on the Zoom videoconference platform. Post-course evaluation surveys were completed by 138 participants with favorable responses from attendees. Ninety-three percent (129/138) of attendees reported an increase in their understanding of cervical cancer screening guidelines and 91% (126/138) reported an increase in their ability to perform colposcopy. Ninety-four percent of respondents said they planned to do something differently in their practice. A majority of attendees (98%) stated that the virtual experience was the same or better than an in-person course. Future courses are planned for Mozambique, Ethiopia and Nepal, including one in which the practical training will be covered by videos and live demonstrations.

## CONCLUSIONS

Cervical cancer prevention care including screening, diagnosis and treatment of pre-invasive disease and follow-up of women with abnormal test results, is essential to reduce the burden of cervical cancer. A well-trained cadre of clinicians to provide quality screening and effective management of women with abnormal screening tests is critical for success. It is possible to continue training and mentoring despite the restrictions and social distancing requirements during the pandemic. To deliver virtual training effectively there must be coordination with local clinicians, hospitals and government personnel and support from local providers to serve as on-site instructors and supervisors in coordination with specialists joining remotely.

Use of a virtual platform widened the reach of the training in Mozambique, with engagement from a greater number of participants, including those from distant provinces and from other Portuguese-speaking countries. Expansion of virtual trainings in order to continue educating providers in LMICs during the COVID-19 pandemic is planned.

## References

[1] Bray F Ferlay J Soerjomataram I Siegel RL Torre LA Jemal A Global cancer statistics 2018: GLOBOCAN estimates of incidence and mortality worldwide for 36 cancers in 185 countries. CA Cancer J Clin. 2018;68:394-424. 10.3322/caac.21492 30207593

[2] Lorenzoni CF Ferro J Carrilho C Colombet M Parkin DM Cancer in Mozambique: Results from two population-based cancer registries. Int J Cancer. 2020;147:1629-37. 10.1002/ijc.32953 32142162

[3] World Health Organization. Cancer Country Profiles. 2014. Available: http://www.who.int/cancer/country-profiles/moz_en.pdf. Accessed: 20 January 2021.

[4] Salcedo MP Lorenzoni C Schmeler KM Working together to eliminate cervical cancer: a partnership across three countries “As mudancas no mundo sao criadas por nos”. Int J Gynecol Cancer. 2019;29:981. 10.1136/ijgc-2019-000372 30948429

[5] Parra S Oden M Schmeler K Richards-Kortum R Rice360 Student T. Low-Cost Instructional Apparatus to Improve Training for Cervical Cancer Screening and Prevention. Obstet Gynecol. 2019;133:559-67. 10.1097/AOG.0000000000003140 30741811 PMC6407823

